# Evaluation of the fuel cell performances of TiO_2_/PAN electrospun carbon-based electrodes

**DOI:** 10.3906/kim-2012-19

**Published:** 2021-06-30

**Authors:** Sema ASLAN

**Affiliations:** 1 Departmentof Chemistry, Faculty of Science, Muğla Sıtkı Koçman University, Muğla Turkey

**Keywords:** Battery, fuel cell, pencil graphite electrode, hydrogen production, electrospinning, recovery

## Abstract

Electrocatalytic effect of the untreated and TiO_2_+polyacrylonitrile (PAN) modified discarded battery coal (DBC) and pencil graphite electrodes (PGE) were evaluated in fuel cell (FC) applications. TiO_2_+PAN solution is coated on PGE and DBC electrodes by electrospinning. According to the FESEM and EDS characterizations, TiO_2_ and PAN nanofibers are found to be approximately 40 and 240 nm in size. TiO_2_+PAN/PGE showed the best FC performances with 2.00 A cm^–2^ current density and 5.05 W cm^–2^ power density values, whereas TiO_2_+PAN/DBC showed 0.68 A cm^–2^ current density and 0.62 W cm^–2 ^power density values. Electrochemical characterizations of PGE and TiO_2_+PAN/PGE electrodes were investigated by cyclic voltammetry and electrochemical impedance spectroscopy. Finally, long-term FC measurement results of developed electrodes exhibited very reasonable recovery values. Along with the comparison of the electrode performances, the recovery of DBCs as electrodes for renewable energy production has been achieved.

## 1. Introduction

Nanoparticle modified nanofibers have been successfully applied in divergent areas such as catalysis [1–3], tissue engineering [4], photochemical applications [5–7], capacitors [8], energy applications [9], membrane electrodes in fuel cells[10], etc... Fuel cells (FC) are environmentally friendly alternative power sources [11] and until now, from solid oxide to microbial FCs, various studies have been reported, which use the advantage of electrospun polymers [10,12–15]. Electrospun polymers exhibit unique electrochemical activity. Besides, nanomaterial supported 1D nanofibers can be used as a useful tool due to their small diameters and highly porous structures. Therefore, they gained attention by the electrochemical researchers for the electrode surface modification studies [16]. 

Polyacrylonitrile (PAN) is the most widely used carbon nanofiber precursor due to its high carbon content [17], besides, it is thermally stable and it provides a highly directable molecular structure [18,19]. Zhang et al. (2020) [20] reported that higher PAN content caused higher permeability and conductivity on FC performance. Padmavathi et al. (2013) [18] used Pt-loaded PAN carbon nanofiber in the FC proton exchange membrane. Also, PAN has a very suitable environment for immobilizing nanomaterials. Its carbon-rich nature can be improved by the addition of electrochemical redox-triggering nanomaterials [21]. There have been reported many nanomaterials modified PAN utilized applications, especially on Li-ion batteries [22] or dye-synthesized solar cells [23]. Although, Pt nanoparticle including PAN nanofibers are very effective catalysts in FC applications [24], here we have suggested an alternative cost-effective and useful way. The catalytic effect on the recovered carbon electrode surface is achieved by the combination of PAN and TiO_2_ nanostructures instead of expensive noble- or semi-noble metal nanoparticles [21–26]. 

TiO_2 _nanostructures are generally preferred towards electrode modifications because of their film-forming capabilities, high surface area, optical transparency, biocompatibility, and good conductivity [27]. Beginning with the first report on the excellent shuttle effect of TiO_2_ nanoparticles on solar cells in dye-synthesized solar cells by O’Regan and Gratzel (1991) [28], researchers quickly improved their work in the optical field. Its superiority in photocatalytic studies has overshadowed its use for other power applications [29]. However, thanks to its mesoporous structure, there is a respectable number of TiO_2_ nanoparticle using FC applications [30–32]. Generally, PAN and TiO_2_ nanostructures are used as carbide derivatives in electrocatalytic applications. [33]. In addition, a comparative study on the catalytic activity and stability of TiO_2_, TiN, and TiC-supported Pt electrocatalysts for the oxygen reduction reaction in the proton exchange membrane fuel cells has been reported by Mirshekari and Shirvanian (2019) [34]. Also, Tański et al. (2017) reported a study on the analysis of the optical properties and the energy band structure of PAN/TiO_2_ nanoparticles in the form of thin composite nanofibrous mats [35]. Similarly, some new materials, such as carbon nanotube/polyaniline composite [36], titanium dioxide/polyaniline composite [37,38], have been used as anodes in MFCs and exhibited high current densities [39].

The use of pencil graphite electrodes (PGE) is very common in electrochemical applications [40,41] among other graphite-based electrodes because of its ease of use and purchase, cost-effectiveness, and wearable electrodes. However, waste recovery for catalytic or energy applications is another developing area [42,43]. Especially, waste battery materials are used for their metal components [44], such as lead [45] or cobalt [46]. Chemical or electrochemical methods are used for the recovery process. Discarded battery coals (DBCs) are often used for previous electrical tests of electrical work with good conductivity and large surface area. Therefore, its components and conductivity still need to be investigated after the battery conditions are used. However, our group has recently published a study on the examination of the TiO_2_+PAN coated discarded battery coal (DBC) electrode as a supercapacitor [47] and also Zr and Ce modified DBC electrodes successfully applied in electrolysis cells [48]. In the supercapacitor application, mostly capacitive properties of the developed electrode were investigated and evaluated as a good candidate for FC applications.

**Figure 1:**
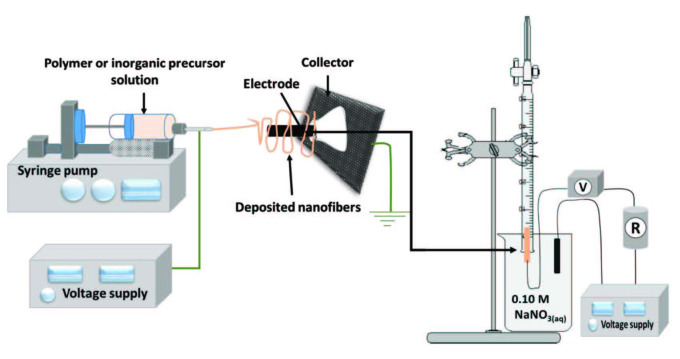
Schematic diagram of the electrospinning setup and FC design.

The electrospinning of DBCs was first investigated in FC applications in the scope of the presented study. The obtained results were found to be significantly improved. Although the pencil graphite electrode (PGE) shows the best FC performances in the presented study, the usage of the DBC is the main innovation of this research and is worth developing. DBC and PGE are specified as carbon-based electrodes. TiO_2_ nanoparticles were suspended in PAN, and electrospinning was performed on the DBC and PGE electrodes (Figure 1).

The characterization of the morphological features of nanoparticles and nanofibers was performed by field emission scanning electron microscopy (FESEM) with energy-dispersive X-ray spectroscopy (EDS), and X-ray diffraction (XRD) measurements. Untreated and TiO_2_+PAN modified DBC and PGE electrodes were used as cathodes to evaluate the electrocatalytic effect of nanoparticle modified nanofibers in FC applications. In addition, the electrochemical characterization of the PGE and TiO_2_+PAN/PGE electrodes was investigated by cyclic voltammetry (CV) and electrochemical impedance spectroscopy (EIS) measurements. Finally, the long-term FC measurement results of the developed electrodes showed reasonable recovery values and found to be promising and practically feasible.

## 2. Experimental

### 2.1. Materials

Titanium (IV) n-butoxide (Ti(OBu)_4_) in isopropyl alcohol (ipa), acetylacetone, nitric acid (HNO_3_) (analytical grade, 99.9%), sodium nitrate (NaNO_3(aq)_), sodium hydroxide (NaOH) (analytical grade, 98% pure), polyacrylonitrile (PAN) (Mw ~ 150,000), and N,N-dimethylformamide (DMF) (99.8%) were purchased from Sigma-Aldrich (Sigma-Aldrich Corp., St. Louis, MO, USA). PGE (Tombow, 0.9 mm) was purchased from a local stationary, DBCs were used from the recovery bins of university (used up Panasonic AA R6 Zinc Carbon 1.5V batteries were used).

### 2.2. Electrospinning system

Figure 1 shows a schematic diagram of the electrospinning of TiO_2_+PAN set up and the apparatus used in this study which consist of a high-voltage power supply, a syringe used as polymer precursor solution reservoir, a syringe pump, collecting plate (covered with aluminum foil), a cone adapter was used for the fixation of electrodes in the upright position to the collector plate. The electrospinning setup is purchased from Inovenso Ltd. Firstly, 10% (w v^-1^) TiO_2_ nanoparticles were suspended in the 10% PAN including DMF solution for 24 h in a sonicator. A positive voltage of 20 kV was applied to the stainless steel needle therewithal to the TiO_2_+PAN polymer solution during the electrospinning process. For grounding, the electrode was connected to DBC or PGE electrodes located on a metallic plate in the upright position (Figure 1) with 15 cm distance. Electrospun fibers were collected at the rate of 0.5 mL s^–1^ on the electrodes by rotating the electrodes manually [2].

### 2.3. Preparation of TiO2 nanoparticles

The TiO_2_ was prepared according to a modified sol–gel method [27] Titanium (IV) *n* -butoxide (Ti(OBu)_4_) in isopropyl alcohol (ipa) solution was used as the precursor of TiO_2_. Acetylacetone (acac) was used to moderate the reaction rate. The molar ratio of the reactants was: Ti(OBu)_4_:H_2_O:ipa:acac = 1:100:2:0.01. Firstly, deionized water was carefully dropwise added to (Ti(OBu)_4_) containing ipa solution for hydrolysis according to the given ratios above. The resulting white precipitate of titanium oxyhydroxide was rinsed by water a couple of times. The final solution was treated with HNO_3_, then refluxed at 85 °C for 8 h up to give a sol (pH ~2.5). Then the sol processed to drying at 100 °C for 3 h in a drying oven, then calcinated in the furnace at 500 °C to give TiO_2_ nanopowder. 

### 2.4. Fuel cell studies

The structure of the FCs was shown in Figure 1. The FC was composed of a 400 mL single-cell compartment, anode, and cathodes. Geometrical surface areas of the cathodes were about 0.22 cm^2 ^and 0.06 cm^2^ for DBC and PGE based electrodes (the geometrical surface areas were calculated according to 2πrh+πr^2^). A multimeter and power supply were utilized for the current-potential readings. A series of resistances ranging from 1 Ω to 10 M Ω were used to obtain polarization graphs. 0.1 M 200 mL of NaNO_3(aq)_ solution was filled into the cell and served as the electrolyte. Measurements were recorded at room temperature and atmospheric pressure. Both the anode and cathode electrodes were immersed into the electrolyte. Electrical connections were provided with crocodiles. Firstly, power (P=IxV) and current (I=V/R) values are calculated then power and current density values are calculated by dividing obtained current values into the geometrical surface area of the cathode electrodes. Obtained power and current densities were plotted vs. potential values obtained from the FC system. Polarization graphics show the maximum power and current density values and the best potential value of the FC systems. FC systems were measured at 3.5 V and 9 V external potentials. All of the experiments were replicated for three times.

### 2.5. Electrochemical measurements

As a result of FC measurements, the best power and current output values were obtained from PGE and TiO_2_+PAN/PGE electrodes. Thus, these electrodes were investigated in terms of the electrochemical activity by CV and EIS methods. Autolab PGSTAT 204 potentiostat/galvanostat (Metrohm Autolab B.V.) electrochemical station equipped with FRA module and driven by NOVA 2.1.4 software was used. PGE or TiO_2_+PAN/PGE were utilized as working electrodes in a three-electrode cell where Ag/AgCl (containing 3 M KCl, CHI115) was the reference and Pt (CH Instruments Inc. CHI 111) served as the counter electrodes in 6 M KOH electrolyte. CV measurements were recorded at the potential range of –0.3 to 0.6 V at the scan rate of 100 mV s^-1^. EIS was measured in a frequency range of 10^–1^ to 10^–4^ Hz in 6 M KOH solution. 

## 3. Results and Discussion

### 3.1. Preparation and characterization of TiO_2_ nanoparticles and TiO_2_/PAN composite fibers

**Figure 2:**
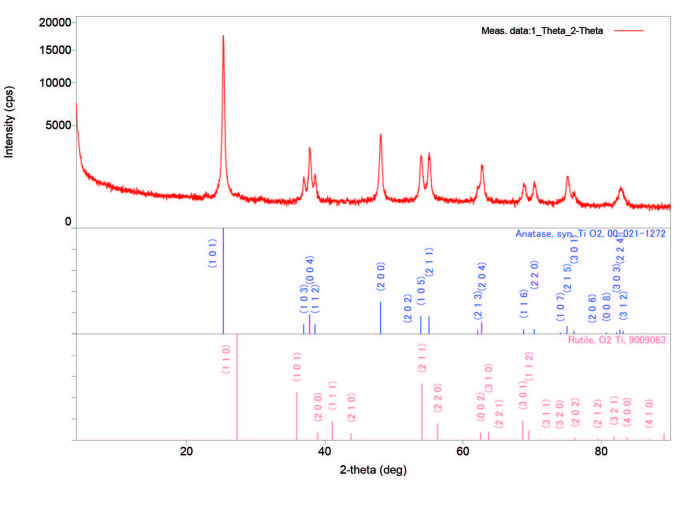
XRD measurements of TiO2 nanoparticles for both anatase and rutile phases.

It has been pointed at the literature that [27] the pH value control is crucial on the obtaining final size of TiO_2_ particles during the process. Titanium (IV) n-butoxide is appointed as an effective precursor for TiO_2_ synthesis since a stable sol can be obtained at the harshly acidic condition at pH < 2. Besides, the heat treatment after preparation is another important parameter and can be adjusted according to the desired final composition and microstructure. Firstly, anatase nucleates occurre as the initial kinetic product. Subsequently, the higher calcination temperature leads to phase transformation from anatase to a more stable rutile phase. The fraction of the rutile phase increases by calcination temperatures [49]. The XRD measurements of TiO_2_ nanoparticles showed typical TiO_2_ peaks related to the anatase, and rutile phases (Figure 2). XRD patterns exhibited strong diffraction peaks at 25° and 48° indicating TiO_2_ in the anatase phase. On the other hand, the peaks observed at 26°, 37° and 55° indicating TiO_2_ in the rutile phase. All peaks are in good agreement with the standard spectrum (JCPDS no.: 88-1175 and 84-1286). The results from XRD indicate that the main phase is anatase but the rutile phase is also observed. Obtained results are in accordance with the given literature [49] at 500 °C measurements. 

**Figure 3:**
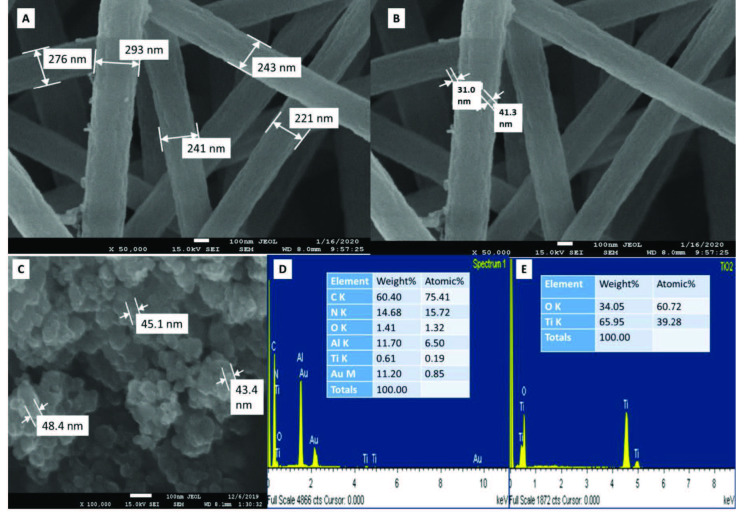
FESEM images of A) fiber size indicated TiO2+PAN, B) the size of the TiO2 indicated in TiO2+PAN, C) synthesized TiO2 nanoparticles before electrospinning, D) EDS spectrum of TiO2+PAN, E) EDS spectrum of TiO2 nanoparticles.

FESEM and EDS results of the synthesized TiO_2_ nanoparticles (Figure 3C, 3B, and 3E) are measured as approximately 40 nm-sized. Atomic and weight percentages are given as inset for both of the PAN+TiO_2_ and TiO_2_ nanostructures. Presence of Al and Au elements in the EDX spectrum of PAN+TiO_2_ are because of the aluminum foil that is used as s collector during electospinning, and, for the imaging of polymeric PAN nanofibers by FESEM, Au coating is needed. The ratios of TiO_2_ nanoparticles are very reliable and consistent for nanofiber encapsulated and natural states. Here, the ratios of the reactants were maintained as reported in the literature, but the calcination temperature was taken as average (500 °C).

Fundamentally the electrospinning is an advanced process that utilizes high DC voltage between a capillary and a conductive surface for the production of delicate nanofibers. In the process a specific electric field is applied to the system when this voltage overcomes the surface tension of the polymer droplet, the polymer solution is charged and ejected as nanofibers are collected on the conductive target. Generally reported PAN-based carbon nanofiber diameters are around 250 nm, although there are lower diameters reached by the usage of DMSO solution [17,50]. Presented nanofibers are synthesized in the range of 230 nm (Figure 3) in the putative interval for carbon nanofibers [9]. Images of the TiO_2_+PAN indicate that (Figure 3) TiO_2_ nanoparticles located on the fiber edges successfully. In EDS spectra (Figure 3 D and E) obtained after electrospinning, the peaks of the Ti element were observed to be compatible with each other. Au peaks are observed because of the coating material of FESEM measurement.

### 3.2. Fuel cell applications

Carbon nanofibers are especially used for battery and other energy applications. Much of these secondary battery studies evaluate the capacitance performance of PAN and its composites with nanoparticles for example, as sodium–selenium batteries [51], long-life sodium-ion batteries [52], Li–S batteries [9]. FCs and batteries diversify excessively, and one of the FC types is voltage induced one [53,54] as exhibited in the presented study. To examine the different voltage inputs for the FCs 3.5 and 9 V, initial voltages were applied to the FC systems. Obtained cell parameters are presented at the consecutive sections.

#### 3.2.1. 3.5 V initial potential applied electrolysis cell measurements

**Figure 4:**
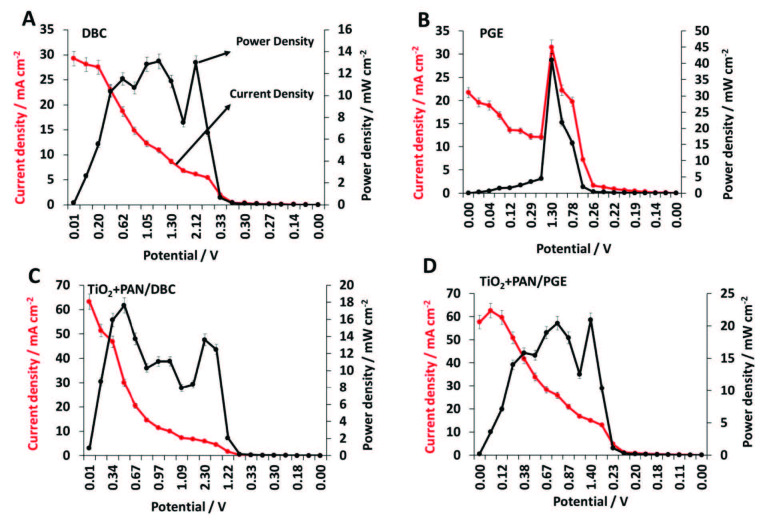
3.5 V measurements of A) DBC, B) PGE, C) TiO2+PAN/DBC, and D) TiO2+PAN/PGE electrodes.

DBC, PGE, TiO_2_+PAN/DBC, and TiO_2_+PAN/PGE electrodes were utilized as cathodes where DBC was employed as the anode in the 0.1 M 200 mL of NaNO_3(aq)_ solution electrolyte filled single-cell FC, and polarisation graphics were obtained. Firstly, DBC cathode was examined and showed 29.39 mA cm^–2^ current density and 13.18 mW cm^–2^ power density values (Figure 4A). Subsequently, PGE cathode showed 31.43 mA cm^–2^ current density and 41.01 mW cm^–2^ power density (Figure 4B), whereas TiO_2_+PAN/DBC showed 63.24 mA cm^–2^ current density and 17.64 mW cm^–2^ values (Figure 4C). Finally, TiO_2_+PAN/PGE electrode showed 62.54 mA cm^-2^ current density and 20.94 mW cm^–2^ power density (Figure 4D). 

When the results are evaluated, it is certain that TiO_2_+PAN nanofiber modification enriched the current and power density values approximately three-fold for DBC and PGE. Besides the results it has been indicated that PGE based electrodes exhibited lower but quite similar current density values with DBC electrodes but slightly reached higher power density values. In comparison, the better FC performance outputs of PGE electrodes that are made of pure graphitic microbeads could be attained to the composite additives in DBC during battery production. These additives may cause an inner resistance compared to a pure graphitic electrode. Apart from these comments, the most important point of this 3.5 potential application experiments is that none of the presented FCs reached to the initial voltage value. Therefore, higher voltage application was examined for upcoming experiments.

#### 3.2.2. 9 V initial potential applied electrolysis cell measurements

**Figure 5:**
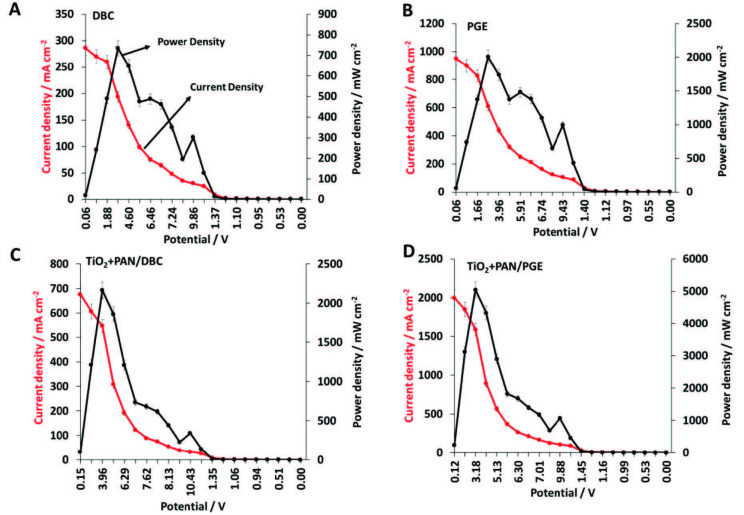
9 V measurements of A) DBC, B) PGE, C) TiO2+PAN/DBC, and D) TiO2+PAN/PGE electrodes.

To further investigate the catalytic effect of the initial charging conditions the voltages between 3.5 and 9 V intervals were examined. Among all, 9 V was found to be applicable and consequent FCs were charged with 9 V. FC systems were measured as mentioned above at 3.5 V measurements. From the polarization graphics, DBC cathode using FC showed 0.29 A cm^–2^ current density and 0.46 W cm^–2^ power density values (Figure 5A). PGE cathode using FC showed 0.95 A cm^–2^ current density and 2.01 W cm^–2 ^power density values (Figure 5B), whereas TiO_2_+PAN/DBC showed 0.68 A cm^–2^ current density and 0.62 W cm^–2 ^power density values (Figure 5C). Later, TiO_2_+PAN/PGE electrode showed 2.00 A cm^–2^ current density and 5.05 W cm^–2 ^power density values (Figure 5E). 

These results, evaluated for both 3.5 V measurements and previous works [24], show that there has been an undeniable improvement on the FC outputs due to the voltage increment. After a successful start-up, the maximum current and power densities for all electrodes were found to be a minimum fifty-fold higher than 3.5 V measurements. It is clear that 9 V provides a positive correlation for the FC system compared to 3.5 V initiated experiments for all cases. Besides, the earliest voltage began to increase strikingly when the FCs were initiated by higher voltage. It reduces the ohmic drop caused by the inner environment of the FC. Additionally, potentials obtained from the whole FC systems reached higher values that indicate the electrodes showed catalytic contribution in the FC systems. Also, the higher current and power density values were recorded at very early stages compared to the 3.5 V measurements, and observed polarization graphics were more stable in terms of voltage drops. Moreover, when the TiO_2_+PAN/DBC and TiO_2_+PAN/PGE electrodes were compared to their bare DBC and PGE electrodes’ results, there have been reasonable increments, respectively. These results suggested that the electrospinning of electrodes with TiO_2_ doped PAN greatly enhance the power generation.

### 3.3. Electrochemical characterization of PGE and TiO2+PAN/PGE

**Figure 6:**
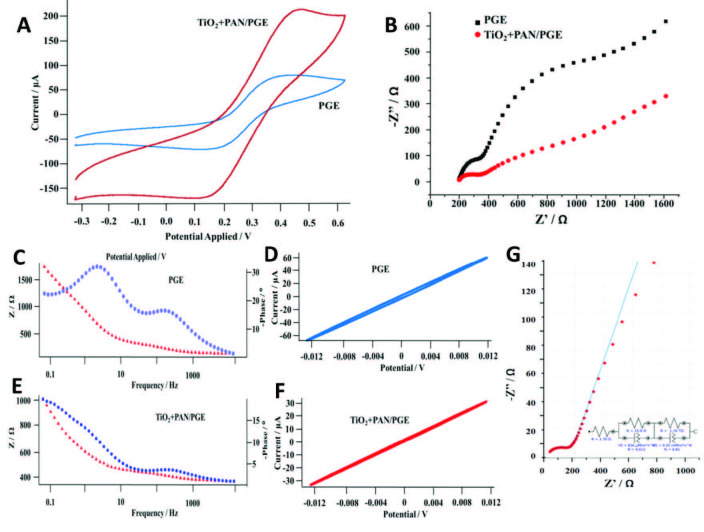
CV and EIS measurements of PGE and TiO2+PAN/PGE electrodes. A) Voltammograms, B) Nyquist plot, C) and E) Bodephase plots, D) and F) Lissajous plots, G) The circuit and the fitting analysis of TiO2+PAN/PGE electrode’s Nyquist plot.

After the impressive progress results on the FC performances of PGE based electrodes, electrochemical activity of untreated and electrospun PGE electrodes was investigated. The CV is generally used to explain the electrochemical mechanism of the electrode with EIS. Here, it is observed that TiO_2_+PAN/PGE electrode showed a cathodic peak position of 0.17 V peak height, 0.06 mA. Anodic peak position 0.36 V; 0.05 mA, these peaks are nearly reversible (Figure 6A). This shows the electron transfer on the electrode surface is in equilibration for both reduction and oxidation. When the peak heights and peak potentials of TiO_2_+PAN/PGE are taken into account cathodic peak position showed a shift to the lower potential as 0.16 V and peak height reached the 0.12 mA value besides, anodic peak position is 0.41 V and the peak height is 0.09 mA. This means that the reduction capacity of the electrode is increased by the electrospinning, it might be because of the excellent supercapacitor feature of PAN [24]. Additionally, TiO_2_ nanoparticles clearly increased the peak current nearly ten folds compared to the results of the PGE electrode (Figure 6A). Of course, these results are needed to be in correlation with impedimetric results. EIS is a reliable technique that has been used to investigate a wide range of experimental systems with very different electrochemical properties [55]. In the given section, EIS measurements of PGE, and TiO_2_+PAN/PGE electrodes are evaluated by three different types of plots named as Nyquist (Figure 6B), bode-phase (Figure 6C and Figure 6E) and Lissajous (Figure 6D and Figure 6F) plots. Nyquist plots are composed of two main parts: semicircle and linear. In an impedance mechanism, if impedance on the electrode surface increases, the semicircle part shows a larger radius but if diffusion of the electrons is higher semicircle gets smaller and linear part shows a higher slope. If the dominant effect in the electrochemical reaction mechanism is diffusion, this type of impedance is called Warburg impedance [56]. Figure 6B shows that TiO_2_+PAN/PGE shows a Warburg type of an impedance spectrum while PGE exhibits two serial semicircles. These results can be evaluated more clearly on Figure 6C and Figure 6E bode plots. Figure 6C and Figure 6E are the bode plots of PGE and TiO_2_+PAN/PGE electrodes, respectively. As the semicircle wanes and linear part increased, diffusion-controlled electron transfer was triggered and conductivity of the electrode increased. These semicircles are observed at PGE based electrode twice while TiO_2_+PAN/PGE based electrode has one semicircle relatively with a smaller radius in accordance with the CV measurements.

The ohmic resistance behaviors of PGE and TiO_2_+PAN/PGE electrodes were also evaluated. The frequency dependence of ΔV value (900 mV) and the corresponding Lissajous plots are shown in Figure 6D and Figure 6F. Figure 6D and Figure 6F are Lissajous plots of PGE and TiO_2_+PAN/PGE electrodes, respectively. As shown in the figures, a sigmoidal response is obtained for a 900 mV input amplitude when the ohmic resistance is large for PGE, whereas a linear response is seen for the TiO_2_+PAN/PGE when the ohmic resistance is small. This result is consistent with Nyquist and bode-phase measurement results [57]. Finally, fitting analysis was conducted to evaluate the surface behavior of the TiO_2_+PAN/PGE electrode (Figure 5G). The best fitting circuit was obtained for [R(RQ)(RQ)] with the lowest estimated error as 0.2 % through fitting, and given as inset in Figure 6G [58]. Here Q denotes either capacitance or resistance in the circuit. It has been seen that hydrophobic nature of PAN nanofiber produced a capacitance or resistance on the surface. Capacitance property of the nanofiber is enhanced by the TiO_2_ nps in the composite structure [47].

### 3.4. Long-term recovery measurements

**Figure 7:**
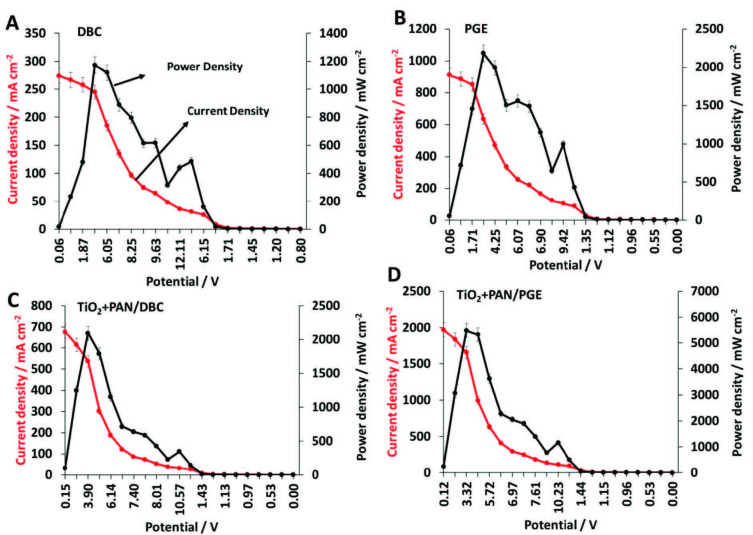
Long-term stability measurements of A) DBC, B) PGE, C) TiO2+PAN/DBC, and D) TiO2+PAN/PGE electrodes.

The long-term stability of the developed electrodes in FC systems was examined after one-month measurements. All of the above-mentioned electrodes were utilized to the EC systems as cathodes in the same conditions with initial measurements, with 9 V loading. Results are given with the recovery values in brackets. Consecutively DBC, PGE, TiO_2_+PAN/DBC, and TiO_2_+PAN/PGE electrodes were utilized as cathodes in EC systems, and polarisation graphics were obtained. DBC cathode showed 0.27 A cm^–2^ current density (96%) and 0.61 W cm^–2^ power density (67%) values (Figure 7A), PGE electrode showed 0.91 A cm^–2^ current density (96%) and 2.18 W cm^–2^ power density (91%) (Figure 7B), whereas TiO_2_+PAN/DBC showed 0.68 A cm^–2^ current density (99.8%) and 0.59 W cm^–2^ power density (96%) values (Figure 7C), and TiO_2_+PAN/PGE electrode showed 1.98 A cm^-2^ current density (99%) and 5.49 W cm^-2^ power density (91%) (Figure 7D). Here the results indicate that even the power and current density of the TiO_2_+PAN/PGE electrode is remarked as the best values, the recoveries of these measurements are lower than other electrodes. This is attributed to the high current and voltage occurrence on this electrode at once, this creates a perturbation on the thousand grade power output values, hence they are still over 90%’s. 

Rauf et al. (2018) [24] reported that generally commercial Pt/C electrocatalyst and the maximum power density could reach to 0.7–1 W cm^−2^ and Ponce de Leon et al. (2006) [59] reported a range of FC studies, in which obtained power outputs are lower of compatible to presented study. These are just a few samples and can be multiplied to give sight to the applicability of the presented FC system for both of the DBC or PGE. Evaluating the final performances of PGE or DBC based electrodes, in sight of the FC operations a standardized and fast responding and high power outputs providing electrode should be preferred meanly PGE based one. Besides, DBC is also promising in terms of power outputs, but it might be explained in detail in terms of the battery components or stable current production capabilities, which can be more useful when investigated in other electrochemical applications.

## 4. Conclusion

Overall, PGE-based electrodes showed a better ten-fold increase in electrochemical activity than DBC for FC applications. Both electrodes exhibited higher FC performance at high voltages, the best results were obtained using TiO_2_+PAN/PGE as 2.00 A cm^–2^ current density and 5.05 W cm^–2^ power density values. After one month of measurements, electrode recoveries for current and power density performances were 99% and 91%, respectively. It has been found that the results can be improved by selecting a suitable conductive polymer for electrospinning. Based on the promising results in terms of this study, the recovery of discarded batteries needs to be explored and expanded with their use in future FC or biosensor studies.
